# Cancer nanomedicine: a review of nano-therapeutics and challenges ahead

**DOI:** 10.1039/d2ra07863e

**Published:** 2023-03-14

**Authors:** M. Joyce Nirmala, Uma Kizhuveetil, Athira Johnson, Balaji G, Ramamurthy Nagarajan, Vignesh Muthuvijayan

**Affiliations:** a Department of Chemical Engineering, Indian Institute of Technology Madras Chennai 600 036 India joycegitz@gmail.com; b Department of Biotechnology, Bhupat and Jyoti Mehta School of Biosciences, Indian Institute of Technology Madras Chennai 600 036 India

## Abstract

Cancer is known as the most dangerous disease in the world in terms of mortality and lack of effective treatment. Research on cancer treatment is still active and of great social importance. Since 1930, chemotherapeutics have been used to treat cancer. However, such conventional treatments are associated with pain, side effects, and a lack of targeting. Nanomedicines are an emerging alternative due to their targeting, bioavailability, and low toxicity. Nanoparticles target cancer cells *via* active and passive mechanisms. Since FDA approval for Doxil®, several nano-therapeutics have been developed, and a few have received approval for use in cancer treatment. Along with liposomes, solid lipid nanoparticles, polymeric nanoparticles, and nanoemulsions, even newer techniques involving extracellular vesicles (EVs) and thermal nanomaterials are now being researched and implemented in practice. This review highlights the evolution and current status of cancer therapy, with a focus on clinical/pre-clinical nanomedicine cancer studies. Insight is also provided into the prospects in this regard.

## Abstract 2

Approaches to overcome the challenges of cancer nanomedicine drug discovery process.

## Graphical abstract

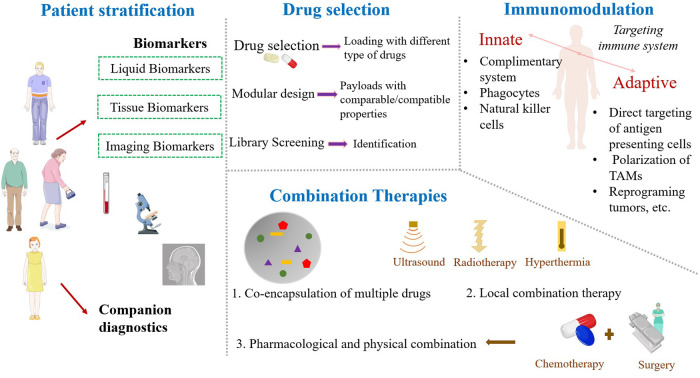

## 1. Introduction

Cancer is a major health concern even today, with the millions of deaths it accounts for every year.^1^ The term “cancer” was first proposed by the Greek physician Hippocrates due to the crab-like appearance of the finger-like projections generated from it.^2^ Another Greek physician, Galen used the term ‘oncos’, which means swelling, to describe tumors.^3^ Cancer has the hallmarks of uncontrolled proliferation, lack of differentiation into mature cells, cell death, altered redox, stress, and metabolic regulation as well as the ability to spread to other parts of the body (metastasis).^4^ These hallmarks of cancer alter the microenvironment of the tumor site, and actively route the energy and nutrient supply for its growth, rather than for bodily functions.^5^ This also results in heterogeneity and mixed populations of cells, each of which has a different response to various therapeutic approaches.^6^

**Fig. 1:**
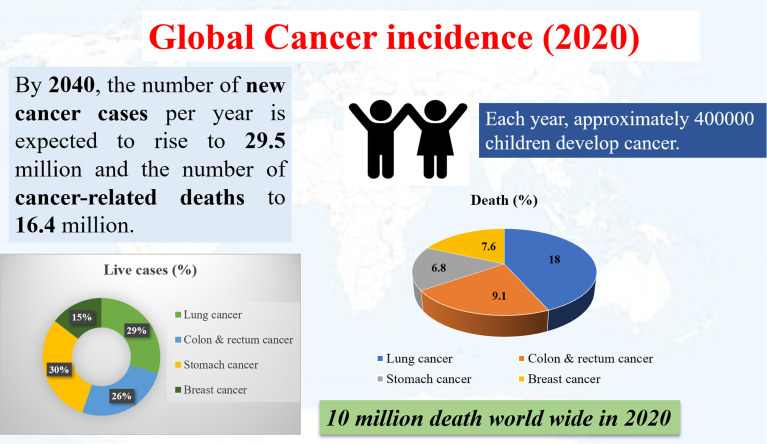
The global incidence of cancer in 2020. Source: World Health Organization.

Many therapeutic methods have been tested against cancer with varying levels of success,^7^ the most common ones being chemotherapy, radiotherapy, and surgery.^8^ Chemotherapy started in the early 1930s with the use of chemicals against tumor cells, prominently with the use of nitrogen mustard against leukemia,^9^ and alkylating drugs such aschlorambucil.^10^ However, the cure for cancer is still evasive, probably owing to the diverse population of cells present in the tumor environment, and the inability of the above-mentioned methods to effectively regulate the tumor microenvironment and the various hallmarks of cancer.^11^ Nowadays, different types of cancer are observed among the population, indicating a high death rate and increasing live cases among children. The mortality rate of several cancers and extrapolated future projections are shown in Fig. 1. According to World Health Organization (WHO), there were 10 million deaths attributable to cancer in 2020, and each year approximately 400 000 children develop cancer. It is expected that by 2040, new cases will rise to 29.5 million with 16.4 million cancer-related deaths.^12^

Nano-oncology is an emerging strategy in cancer therapy that involves the use of nano-dimensional therapeutic materials as anticancer agents, and it has generated promising results in research and clinical trials.^13^ This is a field that has grown tremendously in the past two decades with the development of new approaches not only for cancer treatment but also for diagnosis and prevention.^14^ Nanotechnology has become an innovative method to treat various diseases, owing to its high potential and treatment efficacy in different cancer types. Cancer nanomedicine has wide applications in effective tumor therapy, based on targeting, imaging, viral nanoparticles, and enhanced delivery.^15^

In this review, we present the current status of the research in nano-oncology. Also, a review of promising nano-oncological products in various stages of clinical and preclinical trials is provided.

## 2. Origin and history of cancer nanomedicine

**Fig. 2:**
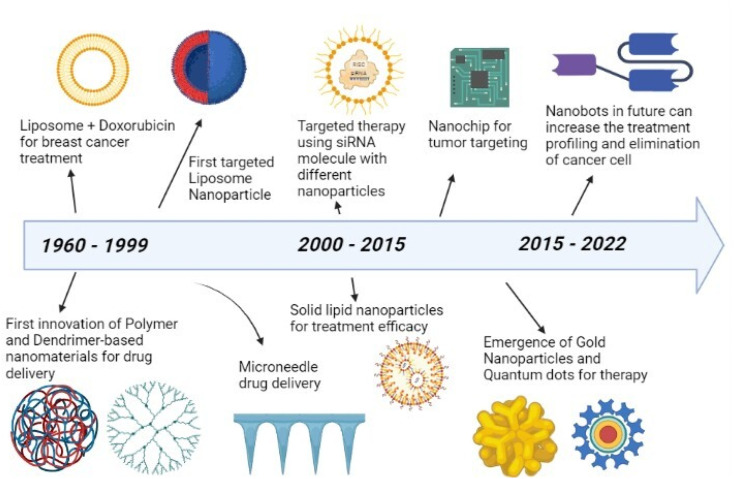
Timeline of cancer nanomedicine.

The early history of nanomedicine may be dated back to ancient times when colloidal gold particles were used for medicinal purposes.^16^ Ancient medical literature has a record of drug preparation methods that use pulverization of medicines to obtain required consistencies, a process later shown to be a nano-scale preparation technique.^17^ Nanomedicine in its current form has been considered a possibility ever since the concept of nanotechnology was first introduced in 1959 by Richard Feynman in his Caltech talk, “There is plenty of room at the bottom”. He mentioned that it would be possible to arrange the atoms as desired. Nanomedicine can be defined as nanotechnology, which deals with the size range of 1 to 100 nm, applied to health and medicine.^18^ In 1999, Robert A Freitas introduced the term “nanomedicine”, and it has been used extensively in technical literature since then. Nanomedicine has been sought due to the deficiencies that exist in treating cancer with drugs of low specificity, rapid drug clearance, biodegradation, and limited targeting.^19^ Nanocarriers were proposed as a superior alternative with targeted drug delivery to the tumor tissue and controlled release of the intended drug.^20^ Nanoparticles or nanomaterials which are innovated and modified are shown over a timeline in Fig. 2. Cancer therapy based on nanoparticles began with the development of doxorubicin-loaded liposomes for treating breast cancer. Later, polymers and dendrimers came into use. Between 2000 to 2015, siRNA molecules with different nanoparticles and solid lipid nanoparticles were developed for targeted therapy and treatment efficacy, respectively.^21^ Then, gold nanoparticles and quantum dots were deployed in cancer therapy, especially for bio-imaging. In the future, it is expected that better treatment can be achieved by using Nanobots.^22,23^

Nanotechnology has enabled applications in a wide range of fields such as Chemistry, Biology, Physics, Engineering, and Medicine. In the clinical industry, it has facilitated the development of delivery systems of drugs for complicated diseases.^24–26^ Nanomedicine is a pioneer field of nanotechnology that deals with the development of powerful techniques for treating diseases and for delivering certain biological compounds for treatment. An injection is the only method of administration for biologics such as peptides, therapeutic proteins, and antibodies (with a few exceptions). Nano-drug delivery has intensified efforts for the development of painless injections, targeted treatment, and an increased ability of the drug to penetrate the BBB (Blood Brain Barrier).^27^ In this century, researchers have advanced the uses of nanotechnology in medicine, with the usage of liposome-mediated drug delivery options, and the encapsulation of drugs such as Doxorubicin and Cabilivi™ as nano-sized drugs. Lipid nanoparticles show promising results in mediating synthetic compounds into nano-drugs.^28^ Loading mRNA and therapeutic proteins as a nano-drug enhances the clinical applications of nanomedicine. Recently, the application of nanomedicine in immunomodulation and immunotherapy has enabled the use of nanoparticles such as acid-base nano-constructs, extracellular vesicles, and virus-modified exosomes.^29^ There are safety concerns with attempts to use nano-medical research in clinical settings, necessitating a better understanding of the crucial characteristics that influence how nanomaterials interact with tissues and organs.^30^

When compared to conventional anticancer therapies, targeted nano-therapy has proved to be a more effective approach with minimum toxicity, enhanced permeability, and retention. It increases the plasma half-life of the nano-size drugs and alters biodistribution, resulting in differential accumulation of nanoparticles in the tumor tissues.^31^ The increased plasma half-life of nanoparticles happens when their size exceeds the limit of the renal excretion threshold and limits their clearance. The differential accumulation of nanoparticles in tumor tissues results in a higher concentration of the nano-sized drug in these tumor tissues than in the plasma or other organs; the accumulation is time-dependent and can be reproduced in tumors of different sizes.^32^ This phenomenon results in prolonged therapeutic effects in addition to targeting when the pharmacological effects and plasma concentration are synergized.^33^

Interest in the application of nanotechnology has increased in cancer therapy because of its advantages in drug delivery, diagnosis, and imaging.^34^ Several therapeutic nanoparticle platforms, such as albumin NPs, liposomes, and polymeric micelles, have been approved for cancer treatment.^35^ Each has its specific characteristics which make them advantageous for certain uses even while posing limitations in certain others.^36,37^

## 3. Mechanism of targeting by nano drug vehicles

A very important criterion for the selection of a nanomedicine formulation for cancer therapy would be its efficiency in targeting the cancer tissue in a specific manner and having minimal side effects on the normal tissue. The various nano-formulations used to deliver anticancer drugs to tumor sites use varying targeting mechanisms for this purpose. The mechanism of drug delivery and the advantages of nanocarriers will vary by carrier. Nanocarriers directly deliver therapeutic agents to the bloodstream and reach the targeted area. They then induce DNA damage by reactive oxygen species (ROS) overproduction. This may finally lead to apoptosis and cell death.^38,39^

**Fig. 3:**
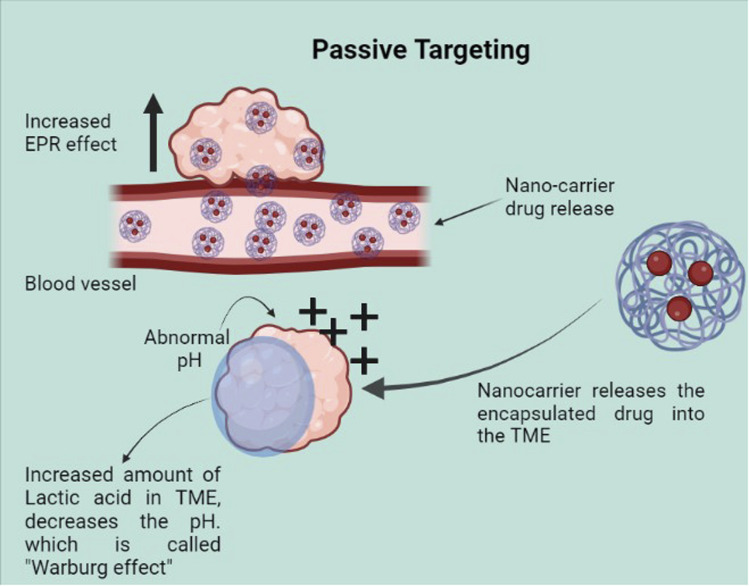
Passive targeting.

Two major types of targeting methods are used for nano-based drug delivery: passive and active. In passive method (Fig. 3), the properties of the tumor site are used to concentrate the nano-vehicles to the tumor site. The major factors used for this are Enhanced Permeability and Retention (EPR) and Tumor Micro Environment (TME) properties. Unlike normal cells, tumor cells induce neovascularisation due to high proliferation and large pores in the vascular walls that favor passive targeting.^40^ Due to imperfect angiogenesis, particles can reach the tumor site and accumulate. Poor lymphatic drainage also increases particle retention resulting in EPR on tumors.^41^ However, the high interstitial fluid pressure inside the tumor microenvironment reduces the uptake and homogeneous distribution of nanoparticles.^42^

**Fig. 4:**
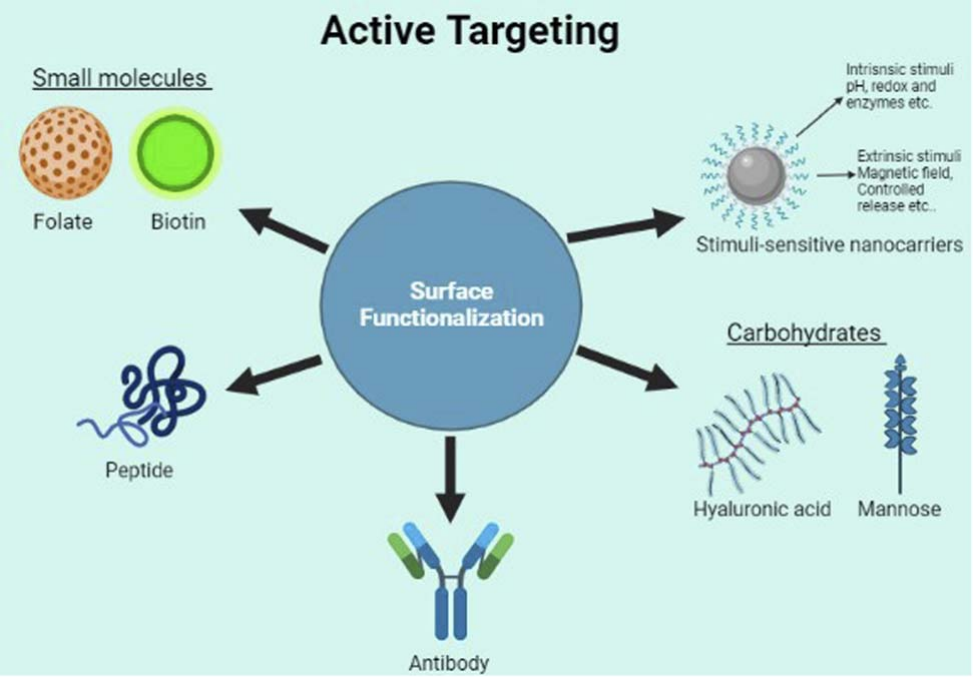
Active targeting.

Although the enhanced permeability and retention effect of tumor tissue causes nanoparticles to preferentially accumulate there to a greater extent than in normal tissue,^43^ the abnormal and dysfunctional tumor microenvironment frequently leads to the heterogeneous distribution of nanoparticles^44^ which primarily reside in the perivascular area and tumor periphery.^45^ Therefore, many nanocarriers also utilize the TME properties such as acidic pH, higher redox potential, and differential secretion of lytic enzymes for uniform drug delivery throughout the tumor. Active targeting (Fig. 4) also utilizes the properties of the tumor cells such as the cell surface receptors expressed by the cancer cells. However, the targeting is achieved by the use of various molecules hybridized along with the carrier to specifically target these. Here, we look into the different modes of targeting used by the various nano-formulations and some of their advantages as well as disadvantages.

In general, passive targeting is based on the diffusion mechanism and it is affected by various factors such as size, shape, and surface properties. It is noted that high bioavailability and reduced renal clearance can be achieved with 40 to 400 nm by increasing the circulation time. Likewise, maintaining the particle size between 50 to 200 and with a rigid and spherical appearance improves the circulation time and also reduces kidney clearance.^46^ Tumor cells were characterized by irregular neovascularization, higher expression of inflammatory factors, and lack of an efficient lymphatic drainage system.^47^ Because of the leaky vasculature structure and poor lymphatic drainage, nanoparticles will eventually enter into tumor tissues and retain them in the tumor bed due to long circulation time. In tumor cells, EPR effect helps to improve drug accumulation, however, in normal tissues, nanoparticles will be cleared by the mononuclear phagocyte system (MPS) or by glomerular filtration of the kidney. Certain barriers such as abnormal tumor vasculature, growth-induced solid stress, and solid stress from the abnormal stromal matrix will hinder the delivery of nanosized drugs.^48^ Gradually, acidic and hypoxic conditions in the tumor cells, heterogeneous perfusion, and elevated interstitial fluid pressure prevent nanoparticle penetration.^49^ These issues can be solved by improving drug delivery by taking the advantage of EPR effect and TME properties.

Maintaining the size of the nanoparticle is better to improve the EPR effect. In addition, the neutral or negative charge of the particles increases the circulation time and drug accumulation by improving plasma half-lives. Applying some adjuvants like nitric oxide donors to improve the EPR effects also can be done.^47^ EPR effect is affected by certain factors such as extravasation, diffusion, and convection in the interstitium, tumor vasculature, and biology, tumor extravascular environment, and physiochemical factors. Also, EPR is heterogenous, in terms of tumor blood flow, hypoxic areas, vascular permeability, extravasation, and penetration.^46^ When the nanoparticles enter the body, it passes through different stages including circulation, endocytosis, accumulation, *etc.* Besides, these particles are highly prone to the opsonization process and because of it; a protein corona will form over the nanoparticles depending on the nanoparticle's characteristics. To avoid this, hydrophilic polymers (PEGylation) with stealth properties can be used to reduce the absorption of opsonins to the nanoparticles. Another strategy is the silencing or depleting of Kupffer cells to escape from the RES system. These cells are specialized macrophages that facilitate the uptake of foreign materials.^50^ Recently, a reduction in tumor hypoxia and modulation of polyethyleneimine cytotoxicity by using PEGylated Prussian blue nanoparticles were reported. It is a dual-enhanced photodynamic therapy with an oxygen self-supply property. Besides, better therapeutic efficacy was observed in breast cancer cells and tumor-bearing mice after laser irradiation.^51^ In another study, evident cell apoptosis and necrosis were observed in PEGylated nanographene oxide-treated cancer cells and this study is an example of combination therapy.^52^ A recently published paper examined an iron-dependent regulated cell death process (ferroptosis) by liposomes embedded with PEG-coated 3 nm γ-Fe_2_O_3_ nanoparticles in the bilayer. These particles promote hydroxyl radical generation and cause efficient lipid peroxidation. The addition of doxorubicin improves the chemotherapeutic effect and traceable magnetic resonance imaging and pH/ROS dual-responsive drug delivery were also visible during the study.^53^

In detail, tumor development is affected by genetic/epigenetic and tumor microenvironment changes (TME). TME consists of tumor cells, stromal fibroblasts, endothelial cells, immune cells, and extra cellular matrix. Disruption of the interaction between stromal cells and tumors is one of the ways to combat tumor progression. Several strategies such as carcinoma-associated fibroblast (CAF) depletion, ECM targeting, anti-angiogenic therapy, exosome/circulating tumor cells (CTCs) targeting, and immune modulation/reprogramming can be done for this purpose.^54^ Designing a pH-responsive drug delivery system is also one of the ways to target tumor cells. Because of the Warburg effect, a large amount of lactic acid will release and it will help the cells to grow in low oxygen and acidic environment. Designing a pH-responsive nanoparticle, which is stable at physiological pH and degradable at tumor pH is the right track to target tumor cells.^55^ Along with this, light-sensitive drug delivery system design is also applicable to cancer treatment. Over expression of matrix metalloproteinase (MMP) is seen in most cancer cells and it can be used as a tumor-specific trigger to tune the size of nanomedicines to enhance tumor penetration. Another possible way is degrading the dense ECM to enhance drug penetration.^56^ Recently, researchers developed gelatin/nanochitosan/doxorubicin nanoparticles for cancer therapy. While reaching the tumor site, MMP-2 degrades gelatin from 178 nm GND to release smaller 4 nm nanochitosan/doxorubicin (ND) nanoparticles for deep tumor penetration and efficient tumor cell endocytosis. Finally, doxorubicin is released due to low pH and MMP-2 activity. In this study, the biocompatibility of the drug was also reported in a mouse tumor bearing model.^57^ An aptamer-decorated hypoxia-responsive nanoparticles (DGL)n@Apt co-loading with gemcitabine monophosphate and STAT3 inhibitor HJC0152 was developed to evaluate its tumor penetration in pancreatic ductal adenocarcinoma cells. This particle can reverse its charge in TME and reduce the size triggered by hypoxia.^58^

Immunotherapy is also considered as a promising technology to treat cancer. This technique involves the modulation of cell-specific immune responses toward the tumor. TME contains immunosuppressive cells like tumor associated macrophages (TAMs). So, cancer therapy has been improved by targeting TAMs.^59^ Solid tumors contain up to 50% of macrophages and it produces cytokines while recruiting to tumor site by the microenvironment. This process may depend on lactic acid level, inflammation, and local anoxia.^60^ M1 (proinflammatory and antitumor) and M2 (anti-inflammatory and pro-tumor) are the two subsets of TAMs^61^ It is noted that the interaction of immune cells with nanoparticles activates the immune system and thereby can improve therapeutic effects. Tumor growth can be reduced by depolarizing the M2 type to the M1 type and this TAM can serve as a drug depot for the accumulation of nanoparticles. So that, local delivery is also achievable.^62^ Recently, the anti-tumor activity of polyethylene glycol-conjugated gold nanoparticles was reported. In this study, nanoparticles suppressed TAMs M2 polarization *via* induction of lysosome dysfunction and autophagic flux inhibition in both *in vitro* and *in vivo* models.^63^ In another study, gene shifts in TAMs towards M1 type and also induced apoptosis in cancer associated fibroblasts were observed upon treating with a biodegradable nanoparticle called ONP-302 during *in vivo* analysis. These particles are negatively charged and were developed from poly(lactic-*co*-glycolic acid) (PLGA).^64^

### 3.1. Liposomes

Made from cholesterol and other natural or synthetic phospholipids,^65^ liposomes are one of the most commercially successful nano-drug delivery platforms.^66^ Both oral delivery and injection methods are applicable in the case of liposome-based drug delivery systems.^67^ FDA-approved intravenous injection is a primary route of administration for various drugs. Besides, subcutaneous, intradermal, intraperitoneal, and intramuscular administrations are also available. The mechanism of liposome drug delivery action relies on the accumulation, uptake, and release of drugs.^68^ The interactions with drug and tumor sites happen *via* passive and active targeting.^69^ Passive targeting mostly uses the enhanced permeability and retention effect observed in tumor microenvironments to improve the retention of liposomes at the tumor sites.^70^ However, active targeting by surface functionalization has improved the drug-targeting ability of liposomes.

Active targeting of liposomes for drug delivery to tumor sites uses surface functionalization of the lipids surface using various methods. The use of antibodies, small molecules, peptides, or carbohydrates has been tested for surface functionalization.^71^ Antibodies, with their specific targeting properties, can be used for targeting cancer-specific surface antigens such as Melanoma cell adhesion molecule (MCAM),^72^ Her 2 receptor, CD44, and growth factor receptors such as VEGFR and EGFR.^71^ Surface functionalization using small molecules such as folate, estrone, and anisamide are also effective in targeting tumor surface receptors for these molecules. Carbohydrates and proteins such as mannose and Beta FGF which can bind to specific cell surface receptors have also been investigated as potential surface active molecules.

Along with surface molecule based targeting, another method used to target liposomes is the stimuli-sensitive cleavage of coating polymers used to reduce the phagocytotic clearance of liposomes. Polymers such as PEG used for coating liposomes increase their circulation and may have reduced targeting properties. To improve the cancer site targeting by these coated liposomes, various stimuli based cleaving have been used. These methods also use the tumor microenvironment properties such as altered pH, redox potential, and secreted enzymes to induce cleavage of the surface coating, thereby inducing preferential drug release at the tumor site.^71^

### 3.2. Extracellular vesicles

Extracellular Vesicles (EVs), released by prokaryotic and eukaryotic cells into the extracellular environment, have recently gained prominence as cancer nano-drug vehicles. EVs may be actively produced by cells in a constitutive or inducible manner, and are naturally nano-scale to micron-sized membrane vesicles encased by phospholipid bilayers.^73^ EVs originate from the endosomal compartment. Their biochemical content consists of lipids, proteins, and microRNA, making them a promising candidate for targeted therapy.

EVs have favorable cellular uptake properties due to their biochemical composition and also can be targeted to tumor sites using surface functionalization.^74,75^ The inherent properties of EVs being able to cross the blood–brain barrier also make them promising drug delivery platforms for brain tumors. Also, engineered EV shows increased pharmacokinetic ability, drug load stability, and targeted therapy; for example, paclitaxel shows potent anticancer effects in a model of Murine Lewis lung carcinoma pulmonary metastases, and also accumulates in cancer cells upon systematic administration, thereby, improving therapeutic outcomes.^76^ However, the non-scalability of EV production is a major challenge to be overcome before it can become an attractive commercial nano-drug vehicle.

### 3.3. Nanoemulsions

Nanoemulsions are water-in-oil or oil-in-water emulsions of nano-dimensions that can be used for drug delivery to tumor sites.^77^ With the usage of generally recognized as safe (GRAS) oils as vehicles, nanoemulsions have the advantage of reduced side effects.^78^ Similar to liposomes, nanoemulsions also can utilize the EPR effect to passively target the tumor site. However, recently surface functionalization of the outer oil portion of the emulsions are being studied to improve the drug targeting to the specific tumor.^79^ Active targeting methods for nanoemulsions also use peptides such as RGD peptide and transferrin, small molecules such as biotin and folate, or certain antibodies specific to tumor surface antigens.^80^ Novel targets such as lysophosphatidic acid receptor is also being studied to improve the drug targeting to specific tumors.^79^

Advantages of nanoemulsions in their versatility for the drug load promoting combinatorial drug use, inherent ability to overcome multidrug resistance, and the potential ability to use the drug itself as the emulsion without the need for a carrier make them interesting choices for cancer therapy.

### 3.4. Dendrimers

Dendrimers are artificial nanocarriers made of radially symmetric arrangements of monomers^81^ with a tree or branch-like appearance.^82^ They have high surface functionalization and targeting properties owing to their versatility of design. As they can be designed specifically to suit each target, dendrimers are one of the nanocarriers with the easiest surface functionalization properties. The ability to precisely regulate the size of the dendrimers also makes it possible for them to be passively targeted to tumor sites *via* EPR. Dendrimers also can be hybridized into other types of nanocarriers, such as encapsulating them in other polymer shells^83^ making it a versatile drug delivery platform for cancer therapy.

Dendrimers have high carrier capacity for the drugs^84^ and can be specifically engineered for various drug release mechanisms. Some of the commonly used ones include variations in the number of terminal groups and the use of degradable spacers and pH-sensitive linkages.^85^ The surface functionalization methods using vitamins, antibodies, peptides, *etc.*, have also been able to overcome the inherent drawbacks of dendrimer-mediated drug delivery such as rapid systemic elimination.

### 3.5. Nanoparticles from inorganic materials

Nanoparticles from inorganic materials are the other kind of drug delivery system widely used for cancer treatment. The commonly used materials for these nanoparticles include gold and silver nanoparticles, carbon quantum dots, carbon nanotubes, metal oxide particles, and mesoporous silica nanoparticles. They are well-known for their precise targeting, good pharmacokinetics profile, encapsulation of poorly soluble drugs, and diagnostic applications. The size and characteristics of nanoparticles are very important when designing drug delivery systems.^86^ Normally, nanoparticles act as a carrier system to incorporate active ingredients, and they release the active compound into the targeted site to induce cell death.

#### 3.5.1. Gold/silver nanoparticles

Nanoparticles of gold and silver are capable of delivering small as well as large drug molecules to the tumor site. The major mode of drug targeting involves the use of EPR as well as tumor micro-environmental properties such as altered redox potential and pH.^87^ Being metallic, they can also be used for hyperthermic treatment using an external heat source such as microwave, post targeting to the tumor site. However, aggregation and higher cytotoxicity to healthy cells by these metal particles reduce their use unless hybridized with certain polymers such as PEG.^88^

#### 3.5.2. Magnetic nanoparticles

Magnetic nanoparticles are used for cancer therapy, especially for hyperthermic treatment including metallic, or metallic shells and oxides. Usually, a magnetic nanoparticle is prepared from pure metal or in combination with polymers. Delivery of magnetic nanoparticles into the targeted site *via* injection using catheters or hypodermic needles has been demonstrated.^89^ A heat source such as a microwave can be used to induce hyperthermia at the specific target, thereby locally increasing the temperature to around 42 °C. Higher temperature leads to the death of cancerous cells due to their leaky vascular nature, without a significant effect on the normal cells. Apart from hyperthermia, magnetic nanoparticles have been used for theranostic, photodynamic therapy, photothermal ablation therapy, biosensors, drug delivery, and MRI imaging^90^ because of the super-paramagnetic feature of iron oxide nanoparticles.^91^

#### 3.5.3. Carbon quantum dots

Carbon quantum dots (CDQs) are fluorescent carbon nanoparticles with imaging and drug delivery applications in cancer.^92^ They have high targeting capabilities due to their ease of surface functionalization, and therefore, can be used with multiple targeting molecules on the surface. The fluorescence properties of CDQs render them highly capable of bio-imaging, adding to their overall theranostic value.^93^ The low toxicity, high biocompatibility, ease of targeting, and added diagnostic functionalities of CDQs make them one of the most promising emerging nano-drug carrier platforms.

### 3.6. Solid lipid nanoparticles (SLN)

SLNs are a colloidal drug delivery system in which the liquid lipid is replaced by solid lipid.^94^ Generally, as the name indicates, solid lipid nanoparticles consist of a solid lipid, emulsifier, and water. Fatty acids, steroids, waxes, triglycerides, and partial glycerides are commonly used as lipids. SLN can be administered through various routes such as parenteral, oral, rectal, ophthalmic, and topical. Solid solution and core–shell models are the two types of SLNs models.^95^ It is observed that SLNs have higher drug release due to their high surface area, and the homogeneous distribution of the drugs will lead to slow release. In addition to this, the high mobility of the drug and crystallization behavior of lipid carriers also facilitates faster release.^96^ SLN is delivered into the targeted area by passive, active, and co-delivery mechanisms. Passive targeting is mainly achieved due to EPR effects, and active targeting is by recognition of receptors or transporters over-expressed on the surface of tumor cells. The co-delivery method is applied by delivering two compounds to the drug delivery system.^97^ Enhanced pharmacokinetics and efficacy against multidrug-resistant cancer cells were observed in cancer cell lines such as MCF-7, A549, and MDA-MB-231 when treated with vorinostat-loaded solid lipid nanoparticles.^98^

## 4. Status of approved drugs and those under clinical trials

Clinical trials are the last stage of drug development wherein the drug formulations are tested on humans to determine their actual efficacy and side effects, to obtain approval for commercial use of the drug formulation.^99^ There are various phases for the clinical trial of a drug and all of them have to be cleared sequentially for the drug to be approved for medical use against the disease. The duration of each phase, the conditions involved, and the number of people the drug is tested on at each phase is decided by the drug regulatory authority. However, there mostly involves four phases of clinical trials before a drug is approved for medical use.

Phase I of a clinical trial involves less than a hundred people and may include healthy people as control groups as this phase is to determine the safety and dosage of the drug. However, for cancer related drug trials, it is mandatory that the group includes people with that particular type of cancer.^99^ After clearing phase I, the drug can enter phase II clinical trials which is conducted on a few hundred people with a particular cancer. The objective of this phase of the clinical trials is to determine the efficacy and side effects. Therefore, it is common to have double blind studies with placebo control groups for this phase. The next phase also is aimed at determining the side effects. However, the focus is on long term and less common side effects and therefore include a larger study group of up to a few thousand people and can go on up to 3–4 years. Upon successful completion of phase III clinical trials, the drug formulation can be approved and may be marketed for medical use. However, the monitoring continues as phase IV wherein any and all adverse reactions reported are investigated for determining the overall safety and efficacy of the drug.

### 4.1. Approved nano-formulations for cancer therapy

Nano-formulations have been marketed for medical use against cancer since early 90's with the polymer–protein conjugate Zinostatin stimalamer^100^ being approved in Japan against hepatocellular carcinoma and the pegylated liposome Doxil® which was marketed as an anti-ovarian cancer drug formulation in the United States of America.^101^ With time, many other types of nano-formulations including liposomes, metal and metal oxide nanoparticles, polymeric micelles, and lipid nanoparticles have been developed and cleared for medical use by multiple agencies all over the world, with many more under various stages of clinical and preclinical trials. Table 1 gives a list of nano-formulations and the drugs used in them, which have already been approved for medical use.

**Table 1:** Approved nanomedicine drugs in the market for cancer treatment

S. No.	Product	Drug	Nanotechnology platform	Cancer type	Approval	Advantages	Toxicity
1	Zinostatin stimalamer	Styrene maleic anhydride neocarzinostatin (SMANCS)	Polymer protein conjugate	Primary unresectable hepatocellular carcinoma	1994 (Japan)	Enhanced accumulation and EPR effect	Slightly toxic (liver dysfunction)
2	Doxil (Caelyx)	Doxorubicin hydrochloride	Pegylated liposome	Ovarian cancer and AIDS-related Kaposi's sarcoma	1995 (FDA)	Prolonged drug circulation time, drug loading, tumor targeting	Long-term use of Doxil® may predispose female patients to oral squamous cell carcinoma
3	DaunoXome	Daunorubicin	Liposome	HIV-related Kaposi sarcoma	1996 (FDA)	No polyethylene coating and slow release into circulation	Adverse cardiac side effects
4	Lipo-Dox	Doxorubicin	Liposome	Kaposi's sarcoma, breast and ovarian cancer	1998 (Taiwan)	Longer half-life, lower clearance, extended blood circulation time, and better tolerance	Mild myelosuppression and other nonhematological toxicities
5	Myocet	Doxorubicin	Liposome	Breast cancer	2000 (EMA)	Less cardiotoxicity and equal anticancer activity	Relative instability
6	Mepact	Muramyl tripeptide phosphatidyl ethanolamine	Liposome	Non-metastatic osteosarcoma	2009 (EMA)	Longer half-life and less toxic	Chills, fever, headache, myalgias, and fatigue
7	Lipusu	Paclitaxel	Liposome	Breast cancer, non-small-cell lung cancer	2013 (EMA)	Modulate paclitaxel toxicity without affecting antitumor activity	Nausea, vomiting, dyspnea, peripheral neuritis
8	NanoTherm	Fe_2_O_3_	Nanoparticles of superparamagnetic iron oxide coated with amino silane	Glioblastoma, prostate, and pancreatic cancers	2013 (EMA)	High blood circulation time and tumor uptake (EPR), heat production under stimulation with EMF, and teranostic properties	Moderate adverse effect
9	Ameluz	5-Aminolevulinic acid	Gel containing 5-aminolevulinic acid, E211, SoyPC, and PG	Superficial and/or nodular basal cell carcinoma	2011 (EMA)	Sustained release and low toxicity	Transient pain and erythema
10	Depocyt	Cytarabine	Liposome	Lymphomatous malignant meningitis	1999 (FDA)	Improve delivery and reduce toxicity	Arachnoiditis and neurotoxicity
11	Genexol-PM	Paclitaxel	Polymeric micelle	Non-small cell lung cancer	2006 (South Korea)	Controlled drug release and targeted drug delivery	Neuropathy, myalgia, and neutropenia
12	Nanoxel	Docetaxel	Polymeric micelle	Breast and ovarian cancers, NSCLC, and AIDS-related Kaposi's sarcoma	2006 (India)	Good efficacy and pharmacokinetic properties with less toxicity	Myalgia, nausea, anemia, paresthesia, alopecia, diarrhea, and vomiting
13	Marqibo	Vincristine	Liposome	Leukemia	2012 (FDA)	No polyethylene coating and slow release into circulation	Drug toxicity and adverse side effect
14	Onivyde	Irinotecan	Liposome	Pancreatic cancer	2015 (FDA)	Enhanced delivery and reduced systemic toxicity	Diarrhea, nausea, vomiting, neutropenia, and febrile neutropenia
15	Vyxeos	Daunorubicin and cytarabine	Liposome	Acute myeloid leukemia (AML)	2017 (EMA)	Improved overall survival	Febrile neutropenia, fatigue, pneumonia, hypoxia, hypertension, bacteremia, and sepsis
16	Oncaspar	l-Asparaginase	PEGylated conjugate	Acute lymphoblastic leukemia	2006 (FDA)	Improved stability of drug load	Venous thromboembolism, pancreatitis, and hyperglycemia
17	DPH107	Paclitaxel	Lipid nanoparticles	Advanced gastric cancer	2016 (Korea)	Absorption without the need for P-glycoprotein inhibitors or Cremophor EL	Neutropenia and leukopenia
18	NBTXR3 (Hensify)	Hafnium oxide nanoparticles stimulated with external radiation to enhance tumor cell death *via* electron production	Hafnium oxide nanoparticles	Locally advanced squamous cell carcinoma	2019 (CE Mark)	Radiotherapy enhancer	Injection site pain, hypotension, and radiation skin injury
19	Apealea	Paclitaxel	Polymeric micelles	Ovarian, peritoneal and fallopian tube cancer	2018 (EMA)	Improve progression-free survival in combination with carboplatin	Neutropenia, diarrhea, nausea, vomiting, and peripheral neuropathy
20	Ontak	Denileukin diftitox	Recombinant DNA derived cytotoxic protein	Cutaneous T cell lymphoma	1999 (FDA)	Targeted delivery	Acute hypersensitivity-type reactions, asthenia, nausea/vomiting (which led to dehydration in some patients), and infectious
21	Eligard	Leuprolide acetate	Polymeric nanoparticles	Advanced prostate cancer	2002 (FDA)	Controlled release and longer circulation time	Hot flushes followed by malaise/fatigue testicular atrophy, dizziness, gynaecomastia and nausea
22	Abraxane	Paclitaxel	Protein carrier	Various cancers including metastatic and pancreatic cancers	2005 (FDA)	Overcomes very low solubility of paclitaxel	Non-specific binding of paclitaxel to albumin
23	Kadcyla	DM1	Trastuzumab, covalently linked to DM1 *via* the stable thioether linker MCC	HER2+ breast cancer	2013 (FDA, EMA)	High blood circulation time, tumor uptake (EPR), selectivity and less toxicity	Nausea, fatigue, thrombocytopenia, headache, constipation, diarrhea, elevated liver enzymes, anorexia, and epistaxis
24	Pazenir	Paclitaxel	Paclitaxel formulated as albumin bound nanoparticles. Powder for dispersion for infusion	Metastatic breast cancer, metastatic adenocarcinoma of the pancreas, non-small cell lung cancer	2019 (EMA)	High solubility, blood circulation time, tumor uptake (EPR), and less toxicity	Affects non-cancer cells such as blood and nerve cells

Doxil® was the first liposome to get approval in the US in 1995^101^ for the treatment of ovarian cancer and AIDS-related Kaposi's sarcoma. After a year, NeXstar Pharmaceuticals developed daunorubicin-loaded NPs (DaunoXome®) to treat HIV-associated Kaposi sarcoma. In 2000, Myocet® is another formulation that contains doxorubicin and cyclophosphamide and got EMEA approval for treating metastatic cancer. Later, Marqibo® got FDA approval for treating non-Hodgkin's lymphoma and leukemia. In 2013, Lipusu was developed by incorporating paclitaxel for treating gastric, ovarian, and lung cancers.^101^ It is understood that the addition of other compounds such as cholesterol and PEG will help attain the desired properties.^102^ Mifamurtide-loaded liposomes were developed by Takeda Pharmaceutical Limited to treat high-grade non-metastatic osteosarcoma. In 2017, Vyxeos got FDA approval. It is a liposomal formulation of daunorubicin and cytarabine. The investigators experimented on 309 patients with an average age range between 60–75 to treat acute myeloid leukemia (t-AML) or acute myeloid leukemia (AML) with myelodysplasia-related changes (AML-MRC).^103^ Along with it, Irinotecan-loaded PEGylated liposome (Onivyde) and cytarabine-loaded liposome (DepoCyt) also got approval for pancreatic adenocarcinoma and lymphomatous meningitis treatment respectively.^104^

Compared to liposomal preparations, other types of approved nano-formulations are lesser in number. However, the focus on the other types of nano-formulations have also been present and a few are approved for clinical use. Styrene maleic anhydride neocarzinostatin (SMANCS) loaded polymer protein conjugate got approval in Japan (1994) for treating renal carcinoma.^105^ Eligard® is a leuprolide acetate-loaded polymeric nanoparticle that got FDA approval in 2002 for prostate cancer.^106^ Another formulation is nanoxel® composed of *N*-isopropyl acrylamide and vinylpyrrolidone monomers and loaded with Doxetaxel. It got approval in India for the treatment of metastatic breast cancer, ovarian cancer, NSCLC, and AIDS-related Kaposi's sarcoma.^107^ Apealea is another drug that got approval from EMA for treating epithelial ovarian cancer, primary peritoneal cancer, and Fallopian tube cancer. It is a paclitaxel-containing polymeric micellar formulation.^108^ Ferucarbotran (carboxydextran coated) and Ferumoxide (dextran) coated were two iron oxide nanoparticles that got approval for cell labeling, especially in the USA.^109^ NanoTherm is an EMA-approved drug for treating glioblastoma, prostate, and pancreatic cancers. It is a nanoparticle of superparamagnetic iron oxide coated with aminosilane. Moderate adverse effects were identified with NanoTherm and it is good to increase blood circulation time and tumor uptake.^110^

### 4.2. Nano-formulations under clinical trials

The number of nano-formulations under clinical trials has been increasing in recent years with many trials in phase II and III at present. Even though liposomes and its hybrids still dominate the trials, there are many metal oxide nanoparticles, carbon quantum dots, polymeric micelles, nanoemulsions and other types of nano-formulations have also been gaining interest (Table 2).

**Table 2:** Nanomedicine drugs under clinical trial for cancer treatment

Name of drug	Active ingredients/drugs used	Nanocarrier/formulation type	Cancer type	Properties/objectives	Status	Reference
Cetuximab nanoparticles	Cetuximab and decorated with somatostatin analogue	Polymeric nanoparticles	Colorectal cancer	Targeting somatostatin receptors	Phase 1	NCT03774680
Nanoxel	Paclitaxel	Nanoparticle	Advanced breast cancer	Pharmacokinetic profile	Phase 1	NCT00915369
Carbon nanoparticles	Carbon nanoparticle	Carbon nanoparticle	Advanced gastric cancer	Harvest lymph nodes after surgery	Phase 3	NCT02123407
BIND-014	Docetaxel	Nanoparticles	KRAS positive or squamous cell non-small cell lung cancer	Second-line therapy	Phase 2	NCT02283320
Lurtotecan liposome	Lurtotecan	Liposome	Advanced or recurrent ovarian epithelial cancer	Chemotherapy	Phase 2 (completed)	NCT00010179
Nab-paclitaxel plus S-1	Nab-paclitaxel and S1	Nanoparticle	Advanced pancreatic cancer	Efficacy and safety first-line treatment	Phase 2	NCT02124317
Fluorescent nanoparticles conjugated somatostatin analog	Somatostatin analog	CdS/ZnS core–shell type quantum dots with carboxylic acid-functionalized (QDs-COOH)	Breast cancer	Bioimaging	Phase 1	NCT04138342
Magnetic nanoparticles	Iron nanoparticle	Nanoparticle	Prostate cancer	Magnetic thermoablation	Early phase 1 (completed)	NCT02033447
Lipodox®	Doxorubicin hydrochloride	Liposome	Breast cancer and ovarian cancer	Bioequivalence study	Phase 1	NCT05273944
ABI-007 and gemcitabine	Gemcitabine and paclitaxel protein-bound particles for injectable suspension (albumin-bound)	Nanoparticle	Metastatic breast cancer	Combination chemotherapy	Phase 2	NCT00110084
AGuIX gadolinium-based nanoparticles	AGuIX	Gadolinium based nanoparticle	Centrally located lung tumors and pancreatic cancer	Safety and efficacy, stereotactic magnetic resonance-guided adaptive radiation therapy	Phase 1 & 2	NCT04789486
ABI-007	Paclitaxel albumin-stabilized nanoparticle formulation	Nanoparticle	Stage IV non-small cell lung cancer	Safety and tolerance	Phase 1& 2	NCT00077246
Paclitaxel albumin-stabilized nanoparticle formulation and carboplatin	Carboplatin	Nanoparticle	Stage IIIB, stage IV, or recurrent non-small cell lung cancer	Combinational chemotherapy	Phase 2 (completed)	NCT00729612
Nanotax	Nanoparticulate paclitaxel	Nanoparticles	Peritoneal cancers	Safety, pharmacokinetics, and preliminary efficacy	Phase 1 (completed)	NCT00666991
DOTAP:Chol-FUS1	fus1 gene	Liposome complex	Lung cancer	Gene transfer therapy	Phase 1 (completed)	NCT00059605
Nano-QUT	Quercetin	PLGA-PEG nanoparticles	Squamous cell carcinoma	Therapeutic efficacy	Phase 2	NCT05456022
ABI-007 plus capecitabine	Nanoparticle albumin-bound paclitaxel	Albumin-bound (Nab) paclitaxel nanoparticles	Advanced gastric cancer patients	Efficacy and safety	Phase 2	NCT01641783
Granulocyte-macrophage colony-stimulating factor (GM-CSF) with weekly protein bound paclitaxel (Abraxane™)	Paclitaxel albumin-stabilized nanoparticle formulation	Paclitaxel albumin-stabilized nanoparticle formulation	Advanced ovarian cancer, fallopian tube cancer, or primary peritoneal cancer	Treating for cancers that did not respond to previous chemotherapy, chemoimmunotherapy	Phase 2 (completed)	NCT00466960
ABI-009	Sirolimus albumin-bound nanoparticles	Sirolimus albumin-bound nanoparticles	Advanced PEComa and patients with a malignancy with relevant genetic mutations or mTOR pathway activation	Targeting multiple disease conditions	Approved for marketing	NCT03817515
MR-Linac-SPION	Ferumoxytol	Iron oxide nanoparticles (SPION)	Primary & metastatic hepatic cancers	Radiotherapy	Not mentioned	NCT04682847
CD24-gold nanocomposite	CD24 primer and gold nanoparticle	Gold nanoparticles	Salivary gland tumors	Diagnostic tool, biomarker	Not mentioned	NCT04907422
Liposomal daunorubicin	Daunorubicin	Liposome	Metastatic breast cancer	Chemotherapy	Phase 1	NCT00004207
Albumin-bound paclitaxel (Abraxane) in combination with carboplatin and herceptin	Abraxane	Albumin-bound paclitaxel nanoparticle	Advanced breast cancer	Combination therapy	Phase 2 (completed)	NCT00093145
Abraxane® with or without Mifepristone	Mifepristone	Nab-paclitaxel	Advanced, glucocorticoid receptor-positive, triple-negative breast cancer	Pharmacokinetic studies	Phase 2	NCT02788981
AA NABPLAGEM	Ascorbic acid (AA),nanoparticle paclitaxel protein bound, cisplatin, gemcitabine	Nanoparticle paclitaxel protein bound	Metastatic pancreatic cancer	Combination therapy	Phase 1 and 2 (completed)	NCT03410030
SPIO	Ferumoxtran-10 (USPIO)	Superparamagnetic nanoparticle	Bladder cancer, genitourinary cancer and prostate cancer	Detect cancer in the pelvic lymph nodes or malignant pelvic lymph nodes	Not applicable	NCT00147238
Nab-PTX plus S-1 and sintilimab	Nab-PTX	Nanoparticle albumin-bound-paclitaxel	Stage IIIC gastric cancer	Adjuvant therapy	Phase 1 & 2	NCT04781413
EP0057 in combination with olaparib	EP0057 and olaparib	Investigational nanoparticle–drug conjugate with a camptothecin payload	Advanced gastric cancer and small cell lung cancer	Combination therapy	Phase 2	NCT05411679
CRLX101(NLG207)	CRLX101	Nanoparticle formulation of camptothecin	Advanced non-small cell lung cancer	Safety and activity	Phase 2	NCT01380769
Combination of ABI-007 (Abraxane) and GW572016 (Lapatinib)	Abraxane	Paclitaxel albumin-stabilized nanoparticle formulation	Stage I, stage II, or stage III breast cancer	Combination therapy	Early phase 1	NCT00331630
FOLFIRABRAX	Paclitaxel albumin-stabilized nanoparticle formulation, fluorouracil, leucovorin calcium, and irinotecan hydrochloride	Paclitaxel albumin-stabilized nanoparticle formulation	Advanced gastrointestinal cancer	Reduce spreading of cancer	Phase 1 and 2 (completed)	NCT02333188
TKM 080301	siRNA against the PLK1 gene product)	Lipid nanoparticles	Colorectal, pancreas, gastric, breast, ovarian, and esophageal cancers with hepatic metastases	Safety and effectiveness	Phase 1 (completed)	NCT01437007
NK105	Paclitaxel	Micellar nanoparticle	Breast cancer	Verify the non-inferiority	Phase 3 (completed)	NCT01644890
Magnetic particle-ICG	Magnetic tracers (FerroTrace) and indocyanine green (ICG)	Magnetic nanoparticles	Colorectal cancer	Feasibility of sentinel lymph node (SLN) mapping and safety	Phase 1 & 2	NCT05092750
CPX-1	Irinotecan HCl:floxuridine	Liposome	Advanced colorectal cancer	Combinational therapy	Phase 2 (completed)	NCT00361842
NC-6300	Epirubicin	Nanoparticle	Advanced solid tumors or soft tissue sarcoma	Safety and tolerability	Phase 1 & 2	NCT03168061
CPC634 (CriPec® Docetaxel)	Docetaxel	CriPec® nanoparticles	Platinum resistant ovarian cancer (CINOVA)	Efficacy over cancers that are resistant to prior platinum-based chemotherapy, safety, and tolerability	Phase 2 (completed)	NCT03742713
NanoTherm®	Iron nanoparticles	Iron nanoparticles	Intermediate-risk prostate cancer	NanoTherm ablation	Not applicable	NCT05010759
Doxorubicin loaded anti-EGFR immunoliposomes	Anti-EGFR immunoliposomes and doxorubicin	Liposomes	Solid tumors	Efficacy and safety	Phase 1 completed)	NCT01702129
Carbon nanoparticles	Carbon nanoparticles and indocyanine green	Carbon nanoparticles	Colorectal cancer	Lymph node tracers	Phase 2 and 3	NCT04759820
NBTXR3	Hafnium oxide	Nanoparticles	Locally advanced or borderline-resectable pancreatic cancer	Particle activation by radiation therapy	Phase 1	NCT04484909
Liposomal paclitaxel	Paclitaxel	Liposome	Cancer	Pharmacokinetics	Phase 4	NCT00606515
INT-1B3	microRNA (miR-193a-3p)	Lipid nanoparticles	Advanced solid tumors	Safety, pharmacokinetics, pharmacodynamics, and preliminary efficacy	Phase 1	NCT04675996
NanoPac	Paclitaxel	Sterile nanoparticulate paclitaxel	Lung cancer	Chemotherapy and safety	Phase 2	NCT04314895
Docetaxel-PNP and taxotere	Docetaxel	Polymeric nanoparticle	Advanced solid cancer	Pharmacokinetic study	Phase 1 (completed	NCT02274610
Silicon incorporated with quaternary ammonium polyethylenimine nanoparticles	Silicon	Quaternary ammonium polyethylenimine nanoparticles	Carcinoma of head and neck	Antibacterial activity	Phase 1	NCT01007240
E7389 liposomal formulation	Eribulin	Liposome	Solid tumor	Safety, pharmacokinetics (PK) and efficacy	Phase 1	NCT03207672
OTX-2002	Biscistronic mRNA	Lipid nanoparticles	Hepatocellular carcinoma and other solid tumortypes known for association with the MYC oncogene	Safety, tolerability, pharmacokinetics, pharmacodynamics, and preliminary antitumor activity	Phase 1 & 2	NCT05497453
FF-10832	Gemcitabine	Liposome	Solid tumors	Safety and tolerance	Phase 1	NCT03440450
ELU001	Exatecans and folic acid analogs	Silicon core/polyethylene glycol C'Dot nanoparticle	Solid tumors that over express folate receptor alpha (FRα)	Safety and efficacy	Phase 1 & 2	NCT05001282
NBTXR3	Hafnium oxide	Hafnium oxide-containing nanoparticles	Esophageal cancer	Radiation therapy with concurrent chemotherapy	Phase 1	NCT04615013
Irinotecan liposomeand bevacizumab	Irinotecan sucrosofate	Liposome	Platinum resistant ovarian, fallopian tube, or primary peritoneal cancer	Efficacy and toxicity	Phase 2	NCT04753216
mRNA-NP (nanoparticle) vaccine	mRNA	DOTAP liposome vaccine	Early melanoma	Toxicity and feasibility	Phase 1	NCT05264974
Rexin-G	Gene	Nanoparticle	Sarcoma	Safety and efficacy	Phase 1 & 2 (completed)	NCT00505713
Silica nanoparticles	Silica nanoparticles	Silica nanoparticles	Head and neck melanoma	Bioimaging	Phase 1 & 2	NCT02106598
NL CPT-11	CPT-11	Liposome	Glioma	Safety, tolerance and pharmacokinetics	Phase 1	NCT00734682
Mitoxantrone hydrochloride liposome	Mitoxantrone hydrochloride	Liposome	Platinum-resistant ovarian cancer	Safety and efficacy	Phase 1	NCT04718376
Bevacizumab + doxorubicin hydrochloride liposome	Bevacizumab	Doxorubicin hydrochloride liposome	Breast cancer	Combination therapy	Phase 2 (completed)	NCT00445406
Irinotecan liposome	Irinotecan	Liposome	Advanced pancreatic cancer	Safety and tolerability	Phase 1	NCT04796948
LEP-ETU	Paclitaxel	Liposome	Advanced cancer (neoplasm)	Safety, pharmacokinetics, and anti-tumor effect	Phase 1 (completed)	NCT00080418
LE-DT	Docetaxel	Liposome	Solid tumors	Maximum tolerated dose and dose limiting toxicity, pharmacokinetics, and anti-tumor effect	Phase 1 (completed)	NCT01151384
Topotecan liposomes	Topotecan	Liposome	Small cell lung cancer, ovarian cancer, solid tumors	Safety and tolerability	Phase 1 (completed)	NCT00765973
Liposome doxorubicin	Doxorubicin	Liposome	Desmoid tumor	Efficacy and safety	Phase 3	NCT05561036
FF-10850	Topotecan	Liposome	Merkel cell carcinoma	Tolerance and safety	Phase 1	NCT04047251
Pegylated liposomal doxorubicin	Doxorubicin	Pegylated liposome	Endocrine-resistant breast cancer	Chemo-immunotherapy	Phase 1b	NCT03591276
WGI-0301	Archexin®	Lipid nanoparticle	Advanced solid tumors	Safety, tolerability, and pharmacokinetics	Phase 1	NCT05267899

Several phase I trials using nano-platforms for use as drugs, as well as a combined theranostic platforms have reported positive results. Many of the nano-formulations under trial are for developing effective nanocarriers for established and approved anticancer drugs. More than one study is in phase I trial to test the efficacy of Topotecan-based liposomes for advanced solid tumors (NCT04047251),^99^ small cell lung carcinomas as well as ovarian carcinomas (NCT00765973).^111^ Even though the phase I trials have been completed, no results have been published. Another topoisomerase inhibitor undergoing clinical trials for a liposome mediated delivery is Irinotecan (NCT04796948).^112^ This study looks into the safety and tolerability of Irinotecan in combination with 5FU and oxaliplatin and is in the initial stages of phase I trials. Paclitaxel (NCT00080418)^113^ and Doxetaxel (NCT01151384)^114^ containing liposomes are also under phase I clinical trial as a part of various combinatorial therapy formulations. These drugs are being tested using various other nanocarrier platforms such as nanoparticulate paclitaxel (NCT00915369),^115^, (NCT00666991),^116^ albumin-stabilized paclitaxel (NCT00331630)^117^ and polymeric Doxetaxel nanoparticles (NCT02274610).^118^ Other drugs under PhaseI clinical trials as cancer nanomedicine include Cetuximab polymeric nanoparticles (NCT03774680),^119^ Doxorubicin liposome (NCT05273944),^120^ Daunorubicin liposomes (NCT00004207),^121^ doxorubicin PEGylated liposomes (NCT03591276)^122^ and lipid nanoparticles (NCT05267899).^123^ Another emerging field with many ongoing phase I clinical trials are nanomedicine platforms for imaging as well as for radiation therapy. CdS/ZnS core–shell type quantum dots with carboxylic acid-functionalized (QDs-COOH) are being studied for their bio-imaging capabilities as fluorescent nanoparticles conjugated somatostatin analogue (NCT04138342).^124^ Other metal/metal oxide nanoparticles studies include FerumoxytolIron oxide nanoparticles for hepatic cancers (NCT04682847),^125^ CD24 primer and gold nanoparticle being investigated as a diagnostic tool and biomarker (NCT04907422)^126^ for salivary gland tumors, Ferumoxtran-10 Superparamagnetic nanoparticle (NCT00147238)^127^ for detection of cancer in the pelvic lymph nodes or malignant pelvic lymph nodes and Hafnium Oxide Particle activation by radiation therapy against locally advanced or borderline-resectable pancreatic cancer (NCT04484909).^128^ Other studies with antibody based drugs, miRNAs, and targeted therapy against gene targets such as DOTAP: Chol-FUS1liposome complex gene transfer therapy (NCT00059605),^129^ TKM 080301siRNA lipid nanoparticles against the PLK1 Gene Product (NCT01437007)^130^ and INT-1B3 microRNA (miR-193a-3p) lipid nanoparticles are also in progress at phase I of clinical trials. Most of these studies have not had any reported results so far.

Recently, in phase II clinical trial (NCT02596373)^131^ mitoxantrone hydrochloride liposome (Lipo-MIT) was tested in patients with advanced breast cancer. Lipo-MIT was injected intravenously and the efficacy and safety in terms of objective response rate, disease control rate, and progression-free survival were investigated. An altered toxicity profile was observed with Lipo-MIT treatment with fewer cardiovascular events. But this study was limited due to its small sample size.^132^ In another study, the bioequivalence of a hybrid pegylated liposomal doxorubicin (PLD) hydrochloride injection with reference product Caelyx® was evident in patients with ovarian cancer.^133^ Another study reported promising efficacy and fewer toxic effects in platinum-resistant recurrent ovarian cancer patients treated with a formulation containing apatinib and pegylated liposomal doxorubicin. This was a comparative study conducted with and without the addition of apatinib in pegylated liposomal doxorubicin. However, some side effects such as hypertension and decreased neutrophil and white blood cell count were observed in patients who underwent this combinatorial therapy.^134^ Recently, a phase 1 study of Eribulin liposomal formulation (E7389-LF) was examined for breast cancer treatment. Patients received formulation every three weeks and tumor assessment was conducted once in six weeks. Tolerability and antitumor activities were evident in the investigation. However, adverse events such as neutropenia, leukopenia, and thrombocytopenia were observed in patients.^135^ Good efficacy of combinational drugs containing pembrolizumab, bevacizumab, and pegylated liposomal doxorubicin was observed in ovarian cancer patients. But four patients had palmar-plantar erythrodysesthesia.^136^ Liposomal Gemcitabine (FF-10832) is a stable liposome that was tested for antitumor activity in advanced solid tumor patients. Better efficacy and fewer adverse effects were reported in this study.^137^ ThermoDox® is a thermally sensitive liposome loaded with doxorubicin. This particle is triggered by mild hyperthermia and the quantification of accumulated drug concentration can be examined before and after ultrasound exposure. Results showed that this formulation is better for drug delivery and cell penetration.^138^

The percentage of studies which clear the phase II clinical trials to reach the phase III clinical trials are much lesser as compared to those which reach phase II form phase I. The major reason for this is the higher number of participants, thereby increasing the probability of determining the side effects.^99^ There have been a few studies that had to be dropped at phase II and III due to non-availability of participants as well. Phase III clinical trials have even more stringent requirements and are fewer in number. There are two carbon nanoparticle-based cancer nanomedicines currently under phase III trials (NCT04759820),^139^ NCT02123407).^140^ A paclitaxel micellar nanoparticle (NCT01644890)^141^ and a Doxorubicin liposome formulation (NCT05561036)^142^ are also undergoing phase III trials. Some of the formulations have also reached phase IV and have been approved for marketing (NCT00606515),^143^ NCT03817515).^144^ The numbers of effective and safe nano-formulations clearing clinical trials are still very few and there are still challenges ahead to be cleared before cancer nanomedicine can be utilized to its full potential.

## 5. Conclusion and future challenges

In the past years, nanotechnology has been widely applied in all aspects of science, engineering, and technology, and research & development in this discipline has been intense. Many cancer nanomedicine researchers have investigated largely consistent processes, which include formulation, characterization, *in vitro* proof of concept, and validation of anticancer activity in preclinical trials. Low throughput and weak predictive abilities plague validation efforts. This review has covered the evolution of cancer nanomedicine from its inception to its current state-of-the-art. In the beginning, we explained the concept of “nanotechnology” and its advancement during the next years, especially in the case of cancer nanomedicine. From 1959 to till now, advancement in nanotechnology is visible in each and every field, including clinical areas. Some of the formulations got approval and many *in vitro*, *in vivo*, and clinical studies are ongoing. Increased circulation time, suitable size range, less toxicity, and good drug accumulation made it superior to the conventional method. Nanoparticles can target a cancer cell *via* passive and active targeting. Due to EPR effect and small size, nanoparticles can easily enter into the tumor cells and retain them in the tumor bed due to long circulation time. Besides, targeting TME is also the best way to reduce tumor progression. Different types of nano-formulations such as liposomes, extracellular vesicles, nanoemulsions, gold/silver nanoparticles, magnetic nanoparticles, carbon dots, and solid lipid nanoparticles are investigated for tumor targeting, bio-imaging, and drug delivery. However, new methods/or technologies are always under consideration to overcome the current challenges explained below.

To develop nano-therapeutics of high efficacy with accumulation in the tumor site rather than the normal cells should be the main criteria focused by the researchers. Because of the composite structure of the nanoparticles, it is very difficult to identify their toxicity and there is no proper validated model for checking the nano-bio interactions, especially, nano-immuno interactions. Other two factors hindering the approval are reproducibility and transparency. Apart from that, the higher cost and complexity have added restriction to the translation success. The journey of a drug from its manufacturing stage to the approval stage is very complex and time-consuming. In addition, the advancement in the instrumentation and characterization methods are necessary for the accomplishment of a drug product.^145^ Moreover, choice on the drug selection with a combinatorial regimen for the specific disease over the targeted population should also be considered important. Most of the time biological barriers also cause an imbalance in terms of targeted delivery, permeability and penetration, endo/lysosomal escape, intracellular processing and trafficking, and metastasis.^146^

The lab-scale production of drugs are easier to achieve, but large-scale manufacturing seems to be a challenging task due to limitations in the advanced experimental set-up and non-availability of sufficient information on the scale-up technologies. Also, adverse effects are encountered due to the difficulties observed during the scale-up process and in reproducing the preparation process.^147^ The developed nanomedicines, are in general, screened by FDA and EMA, but the lack of standard operating procedure for the evaluation of nanomedicines makes the task tougher.^148^ Checking the drugs for any changes during every phase of the clinical trials seems crucial in terms of safety and biocompatibility. Moreover, well-defined characteristics and reproducibility of the drug are essential for initiating the clinical trials. Challenges faced during nanomedicine manufacturing include the presence of contaminants, poor quality control, insufficient batch-to-batch variability, chemical instability, biocompatibility, low production yield, scalability complexities, high cost, lack of infrastructure, government regulations and lack of funding.^145,149^

**Fig. 5:**
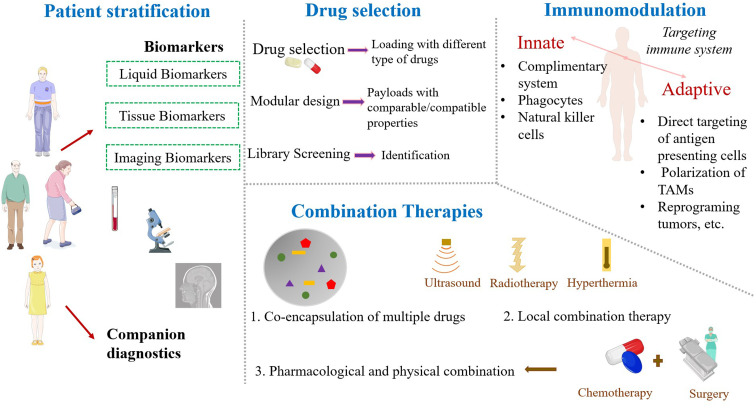
Modern approaches of nanomedicine in cancer therapy. TAMs: tumor associated macrophages. Parts of the figure were drawn using pictures from Servier Medical Art. Servier Medical Art by Servier is licensed under a Creative Commons Attribution 3.0 Unported License (https://creativecommons.org/licenses/by/3.0/).

Patient stratification, drug selection, combination therapies and immunomodulation are some of the important parameters to overcome the challenges of nanoparticles^150^ and these details are illustrated in Fig. 5. Various approaches like CTC analysis, immunohistochemical assessment, and imaging of accumulation of nanomedicines are used for patient stratification.^151,152^ Liquid biomarkers, tissue biomarkers, and imaging biomarkers are currently available. While discussing drug selection, drug classes' modular design and library screening could be considered. Nowadays, combination therapies are widely used in cancer research to increase the efficacy. However, clinical trials for nanomedicines are mostly designed to evaluate monotherapy, which would affect the progress in clinical trials. Improving cancer therapy with the capability to target and modulate the component of the immune system is needed.^146^ A well-designed nanoparticle can be applied as a potential tool for bio-imaging and tumor detection. The high sensitivity of the nano-biosensors is due to the high surface area to volume ratio of the nanoparticle, most preferentially, the gold nanoparticles, quantum dots and polymer dots.^153^ Also, radio-labelled nanoparticles, uniquely suited for positron-emission tomography (PET) or single-photon emission computed tomography (SPECT) mapping of sentinel lymph node and T2-weighted magnetic resonance imaging (MRI) probes are mostly made from iron oxide nanoparticles.^154^ To improve the drug delivery, we have to consider every step ranging from internalization, circulation, penetration into the tumor microenvironment, binding to the target and the destruction of tumor cells. It is necessary to have a clear understanding of the relationship between the physicochemical properties of nanoparticles and their biological response. In recent years, researchers are also focusing on computer algorithms for achieving proper drug delivery.^145^

Opportunities and challenges are two sides of a coin. More clinical trials are needed for the confirmation of preclinical and *in vitro* studies. These should help develop a deep understanding of the interaction of nanoparticles with cells and its after-effects. The main drug delivery routes are oral, intravenous, and subcutaneous for anticancer administration. Inhalation delivery, rectal delivery, and pulmonary delivery are newly proposed. However, these methods are limited due to the high toxicity caused by the combined action of drug deposition and high therapeutic potency. Besides, massive drug doses are needed due to the loss of drugs in the pulmonary tract. In rectal delivery, low absorption is also a major problem.^155^ Recently, mitochondria-based targeting has shown great promise in cancer therapy due to the involvement of mitochondria in tumor generation and progression. Difficulties in clinical trials and bio-safety concerns due to the positive charge on nanoparticles (toxic to normal cells) are the major challenges faced during mitochondrial-targeted therapy.^156^ Another study mentioned that liposomal formulations lose their activity overtime due to their exposure to blood proteins.^157^ The major drawback of carbon-based nanomaterials is the difficulty in standardization due to their non-uniform nature; the phototherapy method is associated with a lack of targeting. Besides, some tumors are difficult to identify due to their small size. Therefore, diagnosis techniques must be improved.^158^

Surely, all the challenges associated with cancer nanomedicine therapy will eventually disappear and, in the future, it will play a crucial role in all aspects of cancer therapy including diagnosis, bio-imaging, and drug delivery.

## Conflicts of interest

The authors report no conflicts of interest in this work.

## Supplementary Material
